# Micro Cellular Rubber (MCR) - a boon for leprosy affected patients with anesthetic feet in preventing secondary impairments

**DOI:** 10.1186/1757-1146-7-S1-A92

**Published:** 2014-04-08

**Authors:** Sathish K Paul, Edward Rajkumar, Tina Mendis

**Affiliations:** 1 Prevention of Impairment and Disability, The Leprosy Mission Trust India, New Delhi-110001, India; 2 Manager, MCR Unit, Vizinagaram, Andhra Pradesh - 531230, India; 3 Head, Sustainable Livelihoods, The Leprosy Mission Trust India-110001, New Delhi, India

## Background

The Micro Cellular Rubber (MCR) unit established in Vizinagaram, Andhra Pradesh in India by The Leprosy Mission Trust India (TLMTI) caters to the need of poor Leprosy affected patients with neuropathic feet within India and to countries nearby India. The MCR Unit till date has provided over a million pairs of MCR insoles to all with anaesthetic feet and still continues to do so.

## Results

The MCR sheets manufactured with a shore hardness of 15' Shore 'A', has helped prevent high pressure points and thus avoid plantar ulcers in anaesthetic feet. Natural Rubber along with several other chemicals is used in optimum quantities to manufacture MCR. The unique manufacturing process gives MCR the ability to spring back to original shape when pressure is released while walking.

The larger size (24” X 20”) coloured MCR sheets with 10mm thickness has become an ideal rubber to prevent stigma for deformed anaesthetic feet. The cost effectiveness in the production of the MCR rubber has helped the poor leprosy patients afford and use the MCR insoles for their footwear. Association and constant interaction with various shoe and footwear companies have led to experimentation and development of newer designs in MCR sandals. High quality and standards of the MCR insoles are maintained through periodic standardised quality tests carried out both within and outside the organisation.

Although the initial purpose of the MCR unit was to cater to the needs of Leprosy affected people, in course of time, various Orthotic & Prosthetic centres realized the value and have started using MCR in their products, especially for Diabetic foot care. Since patients use and prefer protective MCR footwear to prevent ulcers, protect and cover their anaesthetic and deformed feet, it is essential for MCR production units to constantly upgrade and develop newer designs and give the patients and opportunity to choose. At present TLMTI uses 50% of its annual MCR production and the rest is used by other NGOs and Orthotic centres.

## Conclusion

With time there has been a rapid change and development in the design and manufacture of footwear, however there has been no alternative to MCR insole footwear. The constant strive of introducing and making use of MCR footwear in other general disabilities have reduced the stigma of MCR’s in leprosy to a great extent.

